# Ensemble deep learning for Alzheimer’s disease diagnosis using MRI: Integrating features from VGG16, MobileNet, and InceptionResNetV2 models

**DOI:** 10.1371/journal.pone.0318620

**Published:** 2025-04-07

**Authors:** Meshrif Alruily, A A Abd El-Aziz, Ayman Mohamed Mostafa, Mohamed Ezz, Elsayed Mostafa, Ahmed Alsayat, Sameh Abd El-Ghany

**Affiliations:** 1 Computer ScienceDepartment, College of Computer and Information Sciences, Jouf University, Sakaka,Saudi Arabia; 2 Information Systems Department, College of Computer andInformation Sciences, Jouf University, Sakaka, Saudi Arabia; 3 Engineering & ResearchInternational (ERI), Riyadh, Saudi Arabia; Manipal Institute of Technology, INDIA

## Abstract

Alzheimer’s disease (AD) is a neurodegenerative disorder characterized by the accumulation of amyloid plaques and neurofibrillary tangles in the brain, leading to distinctive patterns of neuronal dysfunction and the cognitive decline emblematic of dementia. Currently, over 5 million individuals aged 65 and above are living with AD in the United States, a number projected to rise by 2050. Traditional diagnostic methods are fraught with challenges, including low accuracy and a significant propensity for misdiagnosis. In response to these diagnostic challenges, our study develops and evaluates an innovative deep learning (DL) ensemble model that integrates features from three pre-trained models—VGG16, MobileNet, and InceptionResNetV2—for the precise identification of AD markers from MRI scans. This approach aims to overcome the limitations of individual models in handling varying image shapes and textures, thereby improving diagnostic accuracy. The ultimate goal is to support primary radiologists by streamlining the diagnostic process, facilitating early detection, and enabling timely treatment of AD. Upon rigorous evaluation, our ensemble model demonstrated superior performance over contemporary classifiers, achieving a notable accuracy of 97.93%, along with a specificity of 98.04%, sensitivity of 95.89%, precision of 95.94%, and an F1-score of 87.50%. These results not only underscore the efficacy of the ensemble approach but also highlight the potential for DL to revolutionize AD diagnosis, offering a promising pathway to more accurate, early detection and intervention.

## Introduction

The most common type of dementia, AD, is caused by ongoing harm to memory-related cells. It’s a neurodegenerative disorder that results in a loss of connectivity within the brain, leading to impaired decision-making skills. Gradual death of brain cells reduces memory and cognitive abili-ties and ultimately leads to the inability to perform basic activities like reading, speaking, and writing [1]. The exact cause of AD is unclear, but prevailing theories suggest the accumulation of amyloid beta (A*β*) peptides outside brain cells and a buildup of hyper-phosphorylated proteins within brain cells [2]. Advanced stages of AD may bring about serious complications such as res-piratory issues and heart failure, leading to fatality [3]. Symptoms of AD generally emerge gradu-ally but can intensify over time [4]. The World Alzheimer’s Association states that over 4.8 million Americans aged 65 and older have been diagnosed with AD, predicting that by 2050, 1 in 85 indi-viduals will be affected by AD [5]. Dementia has been classified by the World Health Organization (WHO) as a growing interna-tional health concern. According to predictions, 82 million individuals would have dementia by 2030, up from the current estimated 47 million cases worldwide. Women are more likely than men to get AD symptoms. AD is the second-most severe neurological illness worldwide and is the root cause of 60% to 80% of dementia cases. Assessment of dementia’s severity is often achieved through two widely utilized tools: the global deterioration scale (GDS) and the clinical dementia rating (CDR) scale. The GDS is a straightforward 7-point scale that clinicians commonly used to monitor the disease’s progression, while the CDR provides a more holistic evaluation of an indi-vidual’s cognitive, functional, and behavioral capabilities. Both scales guide families and healthcare providers in making informed decisions regarding treatment and care[6]. The stages of AD are as follows:

- Mild Dementia (MD): Individuals with MD display cognitive impairments that affect their everyday life. They might maintain independent mobility, but may need assistance with certain activities.
- Moderate Dementia (MOD): This is the most extended stage of AD, requiring individuals to need increasing care and support as the disease progresses and daily life becomes more chal-lenging.
- Very Mild Dementia (VMD): This stage is akin to MD. Individuals may retain independent movement but might need help with some activities.
- Severe Dementia (SD): This is the final stage of AD, where individuals lose the ability to communicate and require full-time care.

In AD, the brain typically shrinks, with the cerebral cortex and hippocampus becoming smaller and the ventricles becoming larger [7]. This shrinkage of the hippocampus is associated with im-paired episodic and spatial memory. Neuronal damage in the hippocampus can also lead to communication problems, affecting planning, judgment, and short-term memory [8]. This degen-eration of neurons can eventually lead to synapse damage, neuron death, and further brain shrinkage. Presently, the early stages of AD cannot be promptly and accurately diagnosed due to the limitations of current diagnostic techniques. To improve the accuracy of AD diagnoses, re-searchers are developing new neuroimaging and computer-aided diagnostic (CAD) tools capable of detecting early brain changes associated with AD.

Numerous studies have focused on the early detection and classification of AD. Among the preliminary methods for detecting AD, brain MRI image analysis is the most prevalent. Over the last decade, neuroimaging techniques, particularly Magnetic Resonance Imaging (MRI), have been extensively employed to predict and classify AD [9]. These methods generate detailed, three-dimensional brain images. Typically, machine learning (ML) algorithms for AD classification use manual or semi-manual techniques to extract requisite features from gathered medical images. DL methods have been successfully implemented across multiple domains of medical image analysis, including MRI, ultrasound, mammography, and microscopy [10]. These approaches have yielded significant results in various disease classifications and detections like heart, lung, brain, retina, breast, and bone diseases. However, more work is needed to apply DL techniques for AD detection. Early AD detection through MRI images can help healthcare pro-viders mitigate the disease’s progression in the brain [11].

The current state-of-the-art DL techniques for AD diagnosis using an MRI are aimed at en-hancing sensitivity and specificity as well as early diagnosis. Commonly used for their efficiency in extracting spatial information from MRI images, CNNs have been further extended to Inception-Net, ResNet, DenseNet, etc., which are more efficient. Often, transfer learning is used as well, and the lack of annotated medical data justifies the reliance on practiced models. When multiple models such as VGG16, MobileNet, and InceptionResNetV2, to mention but a few, are used in conjunction, it dramatically improves the classification rates. Furthermore, 3D CNNs and Recurrent Neural Networks (RNNs), especially the Long Short Term Memory (LSTM) networks, involve the struc-tural information of the brain’s three dimensions and the time factor of the progression of the disease. Paying attention increases the model’s focus on the critical areas of the brain; the combi-nation of XAI methods (e.g., Grad-CAM) also contributes to the new model’s interpretability. Multimodal learning fuses MRI with others, and generative models such as GAN enhance the dataset. Frameworks like self-supervised learning enable a model to learn from unlabeled data, and using the best of traditional machine learning and deep learning, diagnostic accuracy can be improved.

This paper introduces a unique ensemble model that combines VGG16, MobileNet, and InceptionResNetV2, aimed at the multi-classification of AD. By fusing features from these models, we aimed to leverage their individual advantages—VGG16’s simple architecture for effective texture recognition, MobileNet’s efficiency and suitability for smaller datasets, and InceptionResNetV2’s deep and complex architecture for capturing fine-grained features. This fusion creates a robust feature set, addressing limitations of single-model approaches, such as overfitting or loss of critical details. All the pre-trained individual feature vectors of VGG16, MobileNet, and InceptionResNetV2 are united into a single feature vector by utilizing a concatenate layer, thus refining the concatenated single feature vector. This potent proposed ensemble model will help healthcare professionals make precise AD diagnoses. The suggested ensemble model could potentially lighten the workload of primary radiologists, enhance early diagnosis, and aid in providing appropriate treatment for AD. The following is a summary of the contributions of this research:

- Proposing a unique ensemble approach, which integrates feature representations from three complementary DL models: VGG16, MobileNet, and InceptionResNetV2. These models were chosen based on their proven performance in feature extraction for medical imaging tasks, each contributing distinct strengths.
- An ablation study was conducted to determine the optimal hyperparameters.
- A comprehensive statistical analysis was carried out across all methods.
- Providing mathematical models for analyzing pre-trained models.
- The proposed ensemble model was evaluated on the MRI image dataset, achieving the following metrics: precision: 95.69%, sensitivity: 95.62%, F1-score: 95.64%, specificity: 98.54%, and accuracy: 97.81%.

The paper continues as follows: Literature Review section presents a literature review on the AD diagnosis system. Materials and methods section delivers a thorough overview of the model’s materials and structure with the proposed ensemble algorithm. Implementation and Evaluation of the Proposed Model section provides details on the implementation and evaluation with a mathematical model for three pre-trained deep learning models and discussions of the results. Finally, Conclusion section gives the conclusion and recommendations for future research.

## Literature review

As presented in [12], a DL model based on the AlexNet architecture, effectively de-tects and classifies AD in MRI images with 91.7% accuracy. It leverages transfer learning by applying a pre-trained CNN to AD image data, achieving high performance through three stages: preprocessing, customization, and potential retraining. This promising ap-proach holds potential for early AD diagnosis and improved patient care. The study by S. Murugan et al. [13] introduces a CNN architecture for AD classification. The model was trained and validated on the Kaggle dataset, commonly used for dementia stage classification. To address class imbal-ance, they employed the SMOTE technique. When tested on a four-class dataset, the model achieved an overall accuracy of 95.23% and an AUC of 97%, demonstrating high accuracy in dis-tinguishing between AD and healthy images. The model identified specific brain regions associated with AD, potentially aiding in creating a decision support system for predicting AD severity. Evaluation on the AD Neuroimaging Initiative (ADNI) dataset yielded an accuracy of 84.83%, suggesting the model’s potential for early AD identification and diagnosis.

The authors of [14] employed four ML algorithms to classify Alzheimer’s Disease (AD) using two datasets: medical records and the Open Access Series of Imaging Studies (OASIS). To address class imbalance, the OASIS dataset underwent balancing with the SMOTE technique, and missing values were replaced using the median approach. Feature connections were calculated, and t-SNE was used to represent high-dimensional data in low-dimensional space. The ML techniques were applied with 20% of the data for testing and 80% for training, with the random forest (RF) classifier outperforming others, achieving an overall accuracy of 94%, along with impressive recall, precision, and F1-score. The second dataset comprised MRI images, which were optimized with an average filter to eliminate noise. Data augmentation prevented overfitting, and a hybrid strategy combining the support vector machine (SVM) algorithm and DL achieved superior results. The AlexNet + SVM model exhibited 93% sensitivity, 94.8% accu-racy, 99.7% AUC, and 97.75% specificity, surpassing the performance of the DL algorithm alone.

In [15], the SRC algorithm was utilized for AD categorization, employing the LMLS-SRC algorithm that incorporated a big margin term and a local constraint term into the conventional SRC model. This modification maintained the multifaceted data structure while ob-taining discriminative dictionary and representation coefficients. The algorithm’s effectiveness was validated on the KAGGLE Alzheimer’s dataset, achieving an 85.54% accuracy in different class classification tasks.

As presented in [16], a new automated system for AD classification based on the AlexNet archi-tecture and convolutional neural networks (CNNs) was proposed. The system, featuring three fully connected layers and five hidden layers, was trained using the Adam optimizer with a learning rate of 0.0001. Tested on an MRI AD image dataset, it achieved a 95% accuracy, 0.1643 loss, and recall, precision, and F1-score values ranging from approximately 91% to 100%.

The authors of [17], presented a two-step method for image classification, involving image filters to generate a numerical dataset and an evolutionary algorithm for classification and knowledge ex-traction. Tested on an AD-related MRI image dataset, the method achieved 100% accuracy for two-class images and 91.49% for three-class images.

As presented in [18], a hybrid classical and quantum machine learning model was proposed for AD identification from MRI scans. Trained on a dataset of 6,400 labeled MRI scans, the model demonstrated significant improvement, achieving 97.2% accuracy on the testing dataset. The hybrid approach integrated classical neural networks and quantum processing, enhancing data pre-processing. The model achieved robust training and classification accuracies of 99.1% and 97.2%, respectively. In a mul-ti-classification scenario, the proposed ensemble deep learning model exhibited superior performance, achieving an accuracy of 95.39%, specificity of 96.32%, sensitivity of 92.48%, precision of 83.89%, and an F1-score of 87.50% compared to prior methods in AD identification from MRI scans.

The limitations of the previous researches are as follows:

1. The authors of the previously mentioned research focused on metrics like accuracy, precision, recall, and F1 score. However, they did not consider evaluating the results using statistical confidence intervals, McNemar’s test, or the P-Value. In comparison, we have successfully employed statistical techniques to analyze and compare the results from various DL methods by examining confidence intervals, applying McNemar’s test, and calculating the P-Value. This highlights the distinct advantage of our approach.
2. The authors of the previously referenced research did not perform an ablation study. In contrast, we conducted this study to understand how individual components or features of our proposed model affect performance. We systematically removed or altered these components and observed the resulting impact on performance.

## Materials and methods

### Data description

The open-source platform Kaggle was used to collect the AD dataset [19], which includes 6400 MRI images from four categories: ND, MD, VMD, and MOD. MRI images dataset was collected by Sarvesh Dubey in 2020. It is a well-structured dataset that is widely used in the research community for tasks related to medical image analysis, particularly in the context of AD diagnosis.

This dataset comprises MRI images of brains, with the objective of distinguishing between the presence and absence of AD, and if present, determining its stage.

The images are typically grayscale, representing different brain regions and their conditions. The images are provided in a standard format (e.g., .jpg or .png), making them easy to use with common image processing libraries and frameworks. While the dataset does not provide details about scanner type, field strength, and imaging parameters, in general, MRI scans used in AD’s research are often acquired using scanners from manufacturers like Siemens, GE, or Philips. The specific model can vary. Field strength is usually either 1.5 Tesla (T) or 3 Tesla (T). Higher field strengths (like 3T) provide better image resolution but can also be more expensive and less comfortable for patients. Imaging Parameters: Voxel Size: from 1mm³ to 1.5mm³ for brain imaging in clinical studies, Repetition Time (TR): from 2,000ms to 3,000ms in T1-weighted imaging, Echo Time (TE): around 30ms to 100ms, depending on the im-aging technique, Slice Thickness: around 1mm to 1.5mm, and Flip Angle: around 12° to 15° in T1-weighted scan.

The MRI dataset contains 176 x 208 images. In our experiment, the MRI images dataset was split into a training set with 5120 images, 80%, and a testing set with 1280 images, 20%. MRI dataset covering various AD stages was used to train the proposed model. With several images from the obtained dataset, the dataset distribution shown in Table 1 makes it abundantly clear that the dataset is class-unbalanced [13].

**Table 1:** The four classes’ distribution in the MRI dataset.

Class	Image Count
ND	3200
MD	896
VMD	2240
MOD	64

The given dataset consists of MRI image of brains and does not pertain any personally identifiable information (PII) such as patient’s name, birthdate, or phone numbers. The images are further erased in a manner that people cannot trace the faces or even some other appendages that would refer them to patients. Typically, in medical imaging datasets like this one, several steps are taken to ensure privacy:

1. De-identification and Anonymization: The images are stripped of all metadata that could reveal the identity of the patient, such as acquisition date, medical record numbers, or any other sensitive information. This is a standard practice in creating medical imaging datasets for research.
2. Ethical Considerations and Consent: While the dataset description does not explicitly mention ethical approvals or consent, it’s generally understood that open-source datasets comply with ethical guidelines and that consent for the use of de-identified data is obtained from patients during the data collection phase. For a dataset collected and shared on platforms like Kaggle, the responsibility of ensuring compliance with privacy regulations typically falls on the data provider.
3. Data Aggregation: The MRI images are aggregated into categories based on Alzheimer’s disease (AD) stages, without any patient-specific information, making it impossible to link the images back to individual patients.

The dataset’s potential biases could influence the fairness and generalizability of the models trained on it through class imbalance. In class imbalance, the dataset description mentions that it is "class-unbalanced," which means that some categories of AD stages (e.g., ND, MD, VMD, MOD) are underrepresented compared to others. This imbalance can lead to a model that performs well on majority classes but poorly on minority ones, impacting the fairness of the diagnostic system. For example, if the dataset has fewer images of mild AD compared to severe AD, the model might not accurately detect the early stages of the disease.

### Model architecture and training

The proposed ensemble model consisted of three distinct DL models: VGG16, MobileNet, and InceptionResNetV2, each selected for its unique architectural strengths and proven efficacy in image classification tasks. VGG16 is renowned for its depth and ability to capture fine details, making it ideal for extracting nuanced features from MRI images. MobileNet, with its lightweight architecture, brings efficiency and adaptability to the ensemble, crucial for handling the varying sizes and resolutions of MRI scans. InceptionResNetV2, combining inception modules with residual connections, offers a sophisticated approach to managing complex patterns in imaging data, crucial for accurate diagnosis. These models, pre-trained on extensive datasets like ImageNet, provide a robust foundation for transfer learning, allowing for effective fine-tuning to the specific requirements of AD diagnosis from MRI scans. Their integration into an ensemble model leverages their complementary strengths, enhancing the overall accuracy and reliability of the diagnostic tool. Additionally, their proven track record in medical image analysis and the balance they offer between computational efficiency and performance make them particularly suited for our study. This ensemble approach aims to harness the best of these models, providing a more comprehensive and accurate diagnostic tool than any single model could achieve.

To detect the AD from MRI scans, the ensemble model was proposed using three DL models: VGG16, MobileNet, and InceptionResNetV2. These DL models were chosen for their high performance in analyzing high-dimensional data such as images. However, each model has its own limitations, such as the inability to handle variations in input image shape and texture. The proposed ensemble model was able to overcome these limitations by combining the strengths of the three models. The proposed ensemble model consists of the following steps:

1. Pre-processing of the MRI dataset: The MRI images are pre-processed to reduce noise and enhance image quality.
2. Splitting the MRI dataset into training and test sets. This was done using the pre-processed MRI pictures.
3. The three deep learning models –VGG16, MobileNet, and InceptionResNetV2–were pre-trained using a sizable image dataset.
4. Combining the pre-trained individual feature vectors: A concatenate layer was used to merge the pre-trained individual feature vectors of VGG16, MobileNet, and InceptionResNetV2 into a single vector of features.
5. Concatenated single feature vector fine-tuning: The training set was used to fine-tune the concatenated single feature vector.
6. Testing the proposed ensemble model: Several metrics, including accuracy, precision, and re-call, were used to test the proposed ensemble model on the test set.

**Fig 1:**
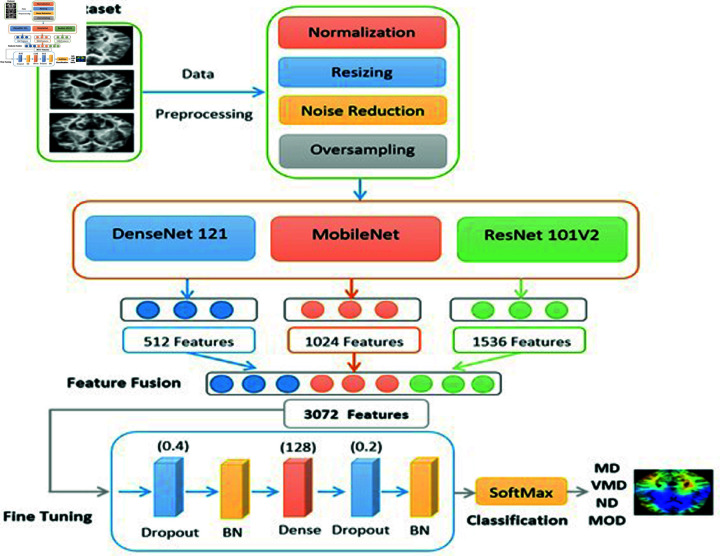
The ensemble architecture of VGGNet16, MobileNet, and InceptionResNetV2.

Algorithm 1 depicts the algorithm of the proposed ensemble model. The steps of the proposed ensemble model are as follows:

On the test set, the suggested ensemble model had a high multi-classification success rate of 95.39 percent. This demonstrates that the suggested model is a viable tool for AD early diagnosis. The ensemble architecture utilized for AD detection is demonstrated in Fig 1.

The pre-processing of MRI image dataset is crucial since it can increase the precision of the outcomes. The pictures were resized and normalized. The following phase involved splitting the dataset into a training set, a testing set, and a validation set. The training of dataset is applied for 80%, 10% is applied for testing, and 10% is applied for validation. The third step was to execute the supervised pre-training phase of the transfer learning process. The VGG16, MobileNet, and In-ceptionResNetV2 models were trained on the ImageNet dataset. All pre-trained models used GlobalAveragePooling2D to flatten all layers into a feature vector by calculating the average value for each input channel. The fourth step was to use a concatenate layer to combine the pre-trained individual feature vectors of VGG16, MobileNet, and InceptionResNetV2 into a single feature vector. The fifth step was to fine-tune the concatenated single feature vector on the MRI images dataset. The combination can capture more useful characteristics, which can improve the model’s accuracy. The proposed ensemble was assessed using measured metrics in the sixth stage.

#### Pre-processing of data

Because real-world data is messy, addressing quality issues in the dataset is a very important part of data pre-processing. Real-world data quality issues include invalid data, missing values, outliers, and duplicate records. As a result, issues must be identified, rectified quality, and addressed. Since data pre-processing affects the results of ML or DL models, data pre-processing of the MRI images dataset was the first step in our experiment. The following techniques were applied to enhance the data quality:

1. **Normalization**: The pixel intensity values of the MRI images were normalized from a range of [0, 255] to [0, 1]. This step helps in reducing the variability in image intensities and stabilizes the convergence during model training by ensuring that the inputs are scaled consistently across all samples.
2. **Resizing**: The images were resized from their original dimensions of 176x208 pixels to 176x176 pixels. Resizing ensures uniform input dimensions across all images, which is essential for batch processing in CNNs.
3. **Noise Reduction**: Gaussian filtering standard noise reduction techniques were implied to enhance image quality by smoothing out noise while preserving edges, which is critical for accurate feature extraction.
4. **Oversampling**: The distribution of the four classes in the MRI images dataset was significantly imbalanced. Specifically, there were 3,200 samples for ND, 896 samples for MD, 2,240 samples for VMD, and only 64 samples for MOD. Many ML algorithms struggle with imbalanced datasets, which can lead to biased training and poor performance on key metrics like accuracy. To address this issue, class balancing was incorporated through over-sampling as part of the data pre-processing. The Synthetic Minority Oversampling Technique (SMOTE) was used to equalize the number of samples across the four classes, resulting in a test set with 639 samples for ND, 662 samples for MD, 635 samples for VMD, and 624 samples for MOD. This brought the total number of samples in the test set to 2,650, ensuring a balanced distribution among the four classes. The distribution of the test set, enhanced by SMOTE, is summarized in Table 2.

**Table 2:** The four classes’ distribution in the MRI dataset after oversampling.

Class	Image Count
ND	639
MD	662
VMD	635
MOD	624

#### VGG16

In 2014, VGGNet was introduced by Karen Simonyan and Andrew Zisserman from the University of Oxford’s Visual Geometry Group (VGG). In the ImageNet Large Scale Visual Recognition Challenge (ILSVRC), VGGNet took first place. With 13 convolutional layers, 3 fully linked layers, and a total of 138 million parameters, it is a convolutional neural network (CNN). VGGNet uses small 3  ×  3 convolution filters, which makes it easier to train and understand. There are five configurations of the VGGNet architecture, labeled A-E [20]. The pre-trained VGG16 architecture has five blocks, with the first four convolution layers in the first two blocks and the remaining nine convolution layers in the next three blocks. Each block is followed by a max-pooling layer with a 2  ×  2 window size. The ReLU activation function is used in each convolution layer.

VGG16 demands considerable computational resources, including memory and processing capabilities, because of its extensive depth and substantial parameter count. The model consists of approximately 138 million parameters, which makes it memory-heavy and can lead to slower performance during both training and inference. Given its complexity and the sheer number of parameters, training VGG16 can be time-consuming, particularly without high-performance GPUs. Additionally, the large number of parameters increases the likelihood of overfitting, especially when the training dataset lacks sufficient size or diversity.

The mathematical model of VGG16 is represented as follows: Let’s denote the input image as X, and the output as Y. The model can be represented as a sequence of operations:

1. Convolutional Layers: Each convolutional layer applies a set of filters to the input to extract features. Let $W{}^{\text{[l]}}$ be the set of weights, $b{}^{\text{[l]}}$ the bias for the $l{}^{\text{th}}$ layer and f the activation function (commonly ReLU). The output $Z{}^{\text{[l]}}$ of the $l{}^{\text{th}}$ convolutional layer is given by:
  $$\begin{array}{ccc} Z{}^{\text{[l]}}=f(W{}^{\text{[l]}}\times Z{}^{\text{[l-1]}}+b{}^{\text{[l]}}) & & \end{array}$$ (1)
2. Max-Pooling Layers: Max-pooling layers reduce the spatial dimensions (height and width) of the input by taking the maximum value in each region of a specified size. Let p be the pooling size. The output $Z{}^{\text{[l]}}$ of a max-pooling layer is given by:
  $$\begin{array}{ccc} Z{}^{\text{[l]}}=Maxpool(Z{}^{\text{[l-1]}},p) & & \end{array}$$ (2)
3. Fully Connected Layers: The fully connected layers connect every neuron to every neuron in the previous layer. The output $Z{}^{\text{[l]}}$ of a fully connected layer is given by:
  $$\begin{array}{ccc} Z{}^{\text{[l]}}=f(W{}^{\text{[l]}}\cdot Z{}^{\text{[l-1]}}+b{}^{\text{[l]}}) & & \end{array}$$ (3)
4. Softmax Layer (Output Layer for Classification): The Softmax layer converts the raw scores from the final fully connected layer into probabilities for each class. Assuming C is the number of classes and $Z{}^{\text{[l-1]}}$ is the output of the last fully connected layer; the final output Y is given by:
  $$\begin{array}{ccc} Y=SoftMax(Z{}^{\text{[l-1]}}) & & \end{array}$$ (4)

#### MobileNet

In order to be used in embedded and mobile vision applications, the CNN-like MobileNet was developed. Condensed design and depth-wise separable convolutions are used in this lightweight CNN. A depth wise layer and a pointwise layer can be used to separate a convolution type known as depth wise separable convolutions. The pointwise layer combines the filtered channels from the depth wise layer to produce the new feature. MobileNet includes 28 layers, and by employing a width multiplier hyperparameters, the number of parameters can be further minimized. The size of the input image is 224 × 224 × 3. Each convolution layer is followed by a ReLU and a batch normalizing layer. Prior to the completely connected layer, the spatial dimension is decreased to 1 by a last average pooling layer. The width multiplier and the resolution multiplier are two different global hyperparameters in the fundamental MobileNet architecture. Each layer’s number of channels is controlled by the width multiplier, while the input image’s size is controlled by the resolution multiplier. These hyperparameters can be used to successfully lower MobileNet’s computational expense without compromising accuracy [21].

When compared to larger and more intricate models like ResNet or Inception, MobileNet typically shows lower accuracy. This compromise arises from its emphasis on efficiency and lower computational demands. The fewer parameters and shallower architecture can restrict the model’s capability to recognize complex features and patterns, particularly in more complicated datasets or tasks that require high accuracy. MobileNet might also face challenges when dealing with high-resolution images due to its lightweight design. The use of depthwise separable convolutions and a smaller number of parameters can impact its effectiveness in processing detailed information. With a reduced number of parameters, MobileNet models may be at a higher risk of overfitting, especially with smaller datasets. To address this issue, it is essential to implement regularization methods and apply careful data augmentation.

The mathematical model of the MobileNet can be described as follows: Let X represents the input, $W{}_{\text{depthwise}}$, $W{}_{\text{pointwise}}$, $W{}_{\text{fc}}$ represent the weights, and $b{}_{\text{fc}}$ represents the bias of the fully connected layer.

1. Depthwise Separable Convolution: The key component of MobileNet is the depthwise separable convolution, which consists of two steps:
  $$\begin{array}{ccc} DepthwiseConvolutionZ{}_{\text{depthwise}}=Depthwise\text{\_}Conv(X,W{}_{\text{depthwise}}) & & \end{array}$$ (5)
  $$\begin{array}{ccc} PointwiseConvolutionZ{}_{\text{pointwise}}=Pointwise\text{\_}Conv(Z{}_{\text{depthwise}},W{}_{\text{Pointwise}}) & & \end{array}$$ (6)
2. Batch Normalization: Apply batch normalization to the output of the pointwise convolution.
  $$\begin{array}{ccc} Z{}_{\text{normalized}}=BatchNorm(Z{}_{\text{pointwise}}) & & \end{array}$$ (7)
3. ReLU Activation: Apply the rectified linear unit (ReLU) activation function:
  $$\begin{array}{ccc} Z{}_{\text{activated}}=ReLU(Z{}_{\text{normalized}}) & & \end{array}$$ (8)
4. Global Average Pooling: Apply global average pooling to reduce spatial dimensions:
  $$\begin{array}{ccc} Z{}_{\text{pooled}}=Global\text{\_}Avg\text{\_}Pool(Z{}_{\text{activated}}) & & \end{array}$$ (9)
5. Fully Connected Layer (Softmax for Classification): Apply a fully connected layer for classification, often followed by the Softmax activation function for multi-class classification:
  $$\begin{array}{ccc} Y=Softmax(W{}_{\text{fc}}\cdot Z{}_{\text{pooled}}+b{}_{\text{fc}}) & & \end{array}$$ (10)

#### InceptionResNetV2

The InceptionResNet model combines the residual connections and inception architecture. It has convolution filters of different sizes trained on large number of images to avoid or reduce degradation. The fundamental elements of the InceptionResnetV2 architecture are present in every layer before the fully connected (FC) layer. The patch size is the kernel size of the FC, pool, or convolution (Conv) layer. The stride is the distance between two consecutive operations. Filter concatenation is a module that combines multiple Conv layers. A network categorization function is called Softmax. ResNet-A, ResNet-B, and ResNet-C are the three primary inception modules in the Inception-ResNetV2 model. These modules extract discriminative features from small Conv layers and decrease their parameter density. Conv and pool layers are unique to each module [22].

The integration of Inception modules and residual connections leads to a highly intricate architecture, making it harder to comprehend, implement, and troubleshoot compared to more straightforward models like VGG16 or ResNet. Although residual connections enhance training efficiency, InceptionResNetV2 still demands considerable computational power and memory re-sources. Without high-performance hardware, both training and inference can be sluggish. The model’s extensive number of parameters results in significant storage demands, which can pose challenges for deployment on devices with limited memory, such as mobile or embedded systems. Additionally, due to its complexity and depth, training InceptionResNetV2 can require a significant amount of time, particularly when dealing with large datasets or lacking access to powerful GPUs or TPUs. The large parameter count also raises the potential for overfitting, especially if the training data is not adequately large or diverse. This necessitates careful implementation of regularization and data augmentation techniques to address the issue.

The mathematical model of the InceptionResNetV2 can be described as follows:

1. Stem Block: The network typically starts with a stem block to process the input. The output $Z{}_{\text{stem}}$ of the stem block is presented as follows:
  $$\begin{array}{ccc} Z{}_{\text{stem}}=StemBlock(x) & & \end{array}$$ (11)
2. Inception Modules: The core of InceptionResNetV2 consists of repeated Inception modules. Each module has multiple parallel branches that process the input in different ways, and their outputs are concatenated. For a given Inception module, the output $Z{}_{\text{inception}}$ is presented as follows:
  $$\begin{array}{ccc} Z{}_{\text{inception}}=InceptionModule(Z{}_{\text{previous}}) & & \end{array}$$ (12)
  Where: $Z{}_{\text{previous}}$ is the output of the previous block.
3. Residual Blocks: Residual blocks with skip connections are inserted at various points in the network to facilitate gradient flow during training. For a given residual block, the output $Z{}_{\text{residual}}$ is presented as follows:
  $$\begin{array}{ccc} Z{}_{\text{residual}}=ResidualBlock(Z{}_{\text{previous}}) & & \end{array}$$ (13)
4. Reduction Blocks: Reduction blocks are employed to decrease the spatial dimensions of the feature maps. For a given reduction block, the output Z_reduction_ is presented as follows:
  $$\begin{array}{ccc} Z{}_{\text{reduction}}=ReduactionBlock(Z{}_{\text{previous}}) & & \end{array}$$ (14)
5. Global Average Pooling: is applied to further reduce the spatial dimensions. The pooled output $Z{}_{\text{pooled}}$ is presented as follows:
  $$\begin{array}{ccc} Z{}_{\text{pooled}}=GlobalAvgPool(Z{}_{\text{previous}}) & & \end{array}$$ (15)
6. Fully Connected Layer (Softmax for Classification): Lastly, a fully connected layer is applied for classification. This layer is often followed by a Softmax activation function for multi-class classification. Let $W{}_{\text{fc}}$ represents the weights, and $b{}_{\text{fc}}$ represents the bias of the fully connected layer. The final output will be as follows:
  $$\begin{array}{ccc} Y=Softmax(W{}_{\text{fc}}\cdot Z{}_{\text{pooled}}+b{}_{\text{fc}}) & & \end{array}$$ (16)

#### Ensemble of DL models and fine-tuning method

In ensemble of DL techniques, multiple DL techniques are trained for a specific problem and combining their results. Hence, it combines the benefits of the DL models with the benefits of an ensemble model [23]. Ensemble of DL models is more complex than ensemble of ML models be-cause DL models have millions of hyper-parameters that need much speed and time to train the various base DL models.

In our experiment, features from AD MRI scans were extracted using three DL models: VGG16, MobileNet, and InceptionResNetV2. The ImageNet dataset, which has millions of photos, was initially used to pre-train the models. Each model was fed with 128  ×  128  ×  3 AD MRI pictures after pre-training. InceptionResNetV2 retrieved 1536 features, MobileNet 1024 features, and VGG16 512 features. The layers were then flattened into a vector by all of the pre-trained models using GlobalAveragePooling2D to determine the average value for each of the input channels. The different vectors were then combined into a single vector with 3072 characteristics using the concatenate layer.

In the second phase of the transfer learning, the concatenated single feature vector on the MRI images dataset was fine-tuned by using six layers (dropout, batch_normalization_203, dense, dropout_1, batch_normalization_204, and dense_1). Since our research objective was multi-classification of AD, the final dense layer had four neurons.

In our experiment, a stacking ensemble was performed. A stacking ensemble involves training a meta-model to make predictions based on the outputs of multiple base models. The mathematical representation for the proposed classification stacking ensemble using VGG16, MobileNet, and InceptionResNetV2 can be expressed as follows: Let’s denote the base models as $M{}_{\text{i}}$, where i ranges from 1 to N, and a meta-model denoted by *Meta*. The output of each base model for a given input X is denoted as $P{}_{\text{i,j}}$(X), where j is the class index. The meta-model takes the predictions of the base models as input and outputs the final prediction.

**Base Models**: For each base model $M{}_{\text{i}}$, the prediction $P{}_{\text{i,j}}$(X) is obtained by passing the input X through the base model.

**Meta-Model Input**: The input for the meta-model is a vector containing the predictions of all base models for a given input X:


$$\begin{array}{ccc} Meta-ModelInput=[P{}_{\text{1,1}}(x),P{}_{\text{1,2}}(x),...,P{}_{\text{N,J}}(x)] & & \end{array}$$ (17)

**Meta-Model Output**: The output of the meta-model is the final prediction *Pensemble*,j(X) for each class j:


$$\begin{array}{ccc} Pensemble,j(X)t=Meta[P{}_{\text{1,1}}(x),P{}_{\text{1,2}}(x),...,P{}_{\text{N,J}}(x)] & & \end{array}$$ (18)

Where J is the total number of classes.

## Implementation and evaluation of the proposed model

### Implementation of proposed model

The MRI image dataset was split into three groups for the suggested model’s implementation: 80% (5120 images) for the training set, 10% (640 images) for the testing set, and 10% (640 images) for the validation set. The Kaggle environment served as the setting for the model’s implementation. During the experiment, the transfer learning process was applied. In the supervised pre-training phase, the VGG16, MobileNet, and InceptionResNetV2 were trained over the ImageNet dataset. Each CNN model was executed five times. In each iteration, each model was evaluated by evalu-ating the measured metrics. At the end of the five iterations, each model’s average was calculated for each metric. Hence, the GlobalAveragePooling2D was used by all pre-trained models simultaneously to flatten all layers into a feature vector by calculating the average value for each input channel. Each CNN model’s last fully connected layers was combined and the proposed ensemble model’s final feature vector was developed. Finally, in the fine-tuning phase, the concatenated single feature vector on the MRI images dataset was fine-tuned and implemented five times. In each iteration, it was evaluated by the measured metrics. At the end of the five iterations, the average of each metric for the proposed fine-tuned ensemble model was calculated.

Tables 3, 4, 5, and 6 present the multi-classification results of the five iterations on the testing set of the MRI images dataset after applying the oversampling and the average of the evaluation metrics for the proposed fine-tuned ensemble model, VGG16, MobileNet, and InceptionResNetV2, respectively. The average accuracy of the proposed fine-tuned ensemble model, VGG16, Mo-bileNet, and InceptionResNetV2, was 97.93%, 94.09%, 95.41%, and 91.15%, respectively. In addition, the confidence intervals for the proposed fine-tuned ensemble model, VGG16, MobileNet, and InceptionResNetV2, were [94.70, 97.16], [88.019, 89.22], [89.54, 92.169], and [82.97, 85.60], respectively. This indicates a 95% confidence that the true value lies within the intervals [94.70, 97.16], [88.019, 89.22], [89.54, 92.169], and [82.97, 85.60].

For the proposed fine-tuned ensemble model, the average precision, sensitivity, specificity, F1-score, and accuracy were 95.94%, 95.89%, 98.04%, 96.36%, and 97.93%, respectively. The VGG16 achieved 88.62%, 88.47%, 94.62%, 89.95%, and 94.09% for precision, sensitivity, specificity, F1-score, and accuracy, respectively. The MobileNet achieved an average of 90.86%, 90.85%, 95.57%, 92.21%, and 95.41% for precision, sensitivity, specificity, F1-score, and accuracy, respec-tively. The InceptionResNetV2 achieved averages of 84.29%, 82.44%, 90.84%, 85.33%, and 91.15% for precision, sensitivity, specificity, F1-score, and accuracy, respectively.

Overall, the proposed fine-tuned ensemble model demonstrated exceptional performance, achieving top scores in accuracy, specificity, precision, recall, and F1-score. These strong metrics across various evaluation criteria position the proposed fine-tuned ensemble model as a highly effective candidate for multi-classification tasks.

**Table 3:** The results of the proposed ensemble on the test set of the MRI dataset.

Iteration	Precision (%)	Sensitivity (%)	Specificity (%)	F1-score (%)	Accuracy (%)
1	95.69	95.62	95.64	98.54	97.81
2	96.43	96.42	98.80	96.00	98.20
3	95.22	95.19	98.37	95.25	97.56
4	96.89	96.92	98.96	96.75	98.44
5	95.43	95.30	98.43	95.25	97.66
**Average**	**95.94**	**95.89**	**98.04**	**96.36**	**97.93**
**STDEVP**	0.6281	0.6714	1.2210	1.2246	0.3345
**Confidence Interval**	[97.28 , 98.59]		

**Table 4:** The results of the VGG 16 on the test set of the MRI dataset.

Iteration	Precision (%)	Sensitivity (%)	Specificity (%)	F1-score (%)	Accuracy (%)
1	88.68	88.70	88.65	96.23	94.36
2	88.71	88.65	96.21	89.00	94.34
3	88.41	88.35	96.12	88.25	94.18
4	88.19	87.70	95.86	87.50	93.77
5	89.10	88.92	96.27	88.75	93.83
**Average**	**88.62**	**88.47**	**94.62**	**89.95**	**94.09**
**STDEVP**	0.3070	0.4208	2.9898	3.1835	0.2500
**Confidence Interval**	[93.60 ,94.58]		

**Table 5:** The results of the MobileNet on the test set of the MRI dataset.

Iteration	Precision (%)	Sensitivity (%)	Specificity (%)	F1-score (%)	Accuracy (%)
1	89.96	89.79	89.73	96.57	94.84
2	91.20	91.28	97.08	91.00	95.63
3	90.14	90.20	96.73	90.00	95.10
4	91.44	91.50	97.16	91.50	95.74
5	91.54	91.50	97.17	92.00	95.76
**Average**	**90.86**	**90.85**	**95.57**	**92.21**	**95.41**
**STDEVP**	0.6697	0.7176	2.9260	2.2762	0.3749
**Confidence Interval**	[94.68 , 96.15 ]		

**Table 6:** The results of the InceptionResNetV2 on the test set of the MRI dataset.

Iteration	Precision (%)	Sensitivity (%)	Specificity (%)	F1-score (%)	Accuracy (%)
1	83.90	76.69	75.77	92.13	88.11
2	84.17	84.31	94.75	84.00	92.13
3	83.31	82.99	94.34	82.75	91.54
4	84.96	84.64	94.87	84.75	92.32
5	85.12	83.56	94.45	83.00	91.64
**Average**	**84.29**	**82.44**	**90.84**	**85.33**	**91.15**
**STDEVP**	0.6728	2.9312	7.5363	3.4765	1.5475
**Confidence Interval**	[88.12 , 94.18 ]		

**Fig 2:**
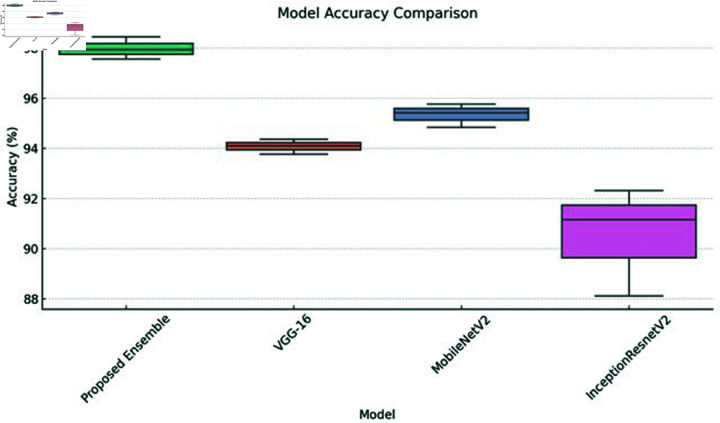
The evaluation of classifiers’ performance using the ANOVA test.

Fig 2 shows the analysis of variance (ANOVA), which is a statistical method used to assess the effectiveness of the proposed fine-tuned ensemble model, VGG16, MobileNet, and InceptionR ResNetV2 classifiers. It allows us to compare the performance of multiple classification models by analyzing the variance among their results.

The performance of the proposed fine-tuned ensemble model, VGG16, MobileNet, and InceptionResNetV2, was evaluated using a boxplot shown in Fig 2. An ANOVA test was conducted on the MRI dataset. The results for the classifiers based on the MRI dataset features revealed the following values: the proposed ensemble model had a lower value of 97.28%, an upper value of 98.59%, and an average of 97.93%; VGG16 had a lower value of 93.60%, an upper value of 94.58%, and an average of 94.09%; MobileNetV2 had a lower value of 94.68%, an upper value of 96.15%, and an average of 95.41%, and InceptionResNetV2 had a lower value of 88.12%, an upper value of 94.18%, and an average of 91.15%.

For the proposed ensemble model, the adjacent minimum and maximum values were 97.28% and 98.59%, respectively, with an average of 97.93%. In the analysis of VGG16, the lower and upper adjacent values were 93.60% and 94.58%, respectively, resulting in a mean value of 94.09%. The MobileNetV2 classifier showed lower and upper adjacent points of 94.68% and 96.15%, respectively, with an average of 95.41%. For the InceptionResNetV2 test, the lower and upper adjacent values are 88.12% and 94.18%, leading to a mean value of 91.15%. The ANOVA results indicate that the proposed ensemble model demonstrated a strong performance on the MRI dataset.

Tables 7, 8, 9, and 10 display the multi-classification results on the testing set of the MRI images dataset for the four classes (ND, VMD, MD, and MOD) using the proposed ensemble model, VGG16, MobileNet, and InceptionResNetV2, respectively. After evaluating the proposed fi-ne-tuning ensemble model, the average precision, sensitivity, F1-score, specificity, and accuracy were 95.69%, 95.62%, 95.64%, 98.54%, and 97.81%, respectively. The VGG16 achieved 88.68%, 88.70%, 88.65%, 96.23%, and 94.36% for precision, sensitivity, F1-score, specificity, and accuracy, respectively. The MobileNet achieved an average of 89.96%, 89.79%, 89.73%, 96.57%, and 94.84% for precision, sensitivity, F1-score, specificity, and accuracy, respectively. The InceptionResNetV2 achieved an average of 83.90%, 76.69%, 75.77%, 92.13%, and 88.11% for precision, sensitivity, F1-score, specificity, and accuracy, respectively.

Tables 7, 8, 9, and 10 provide an in-depth look at the multi-classification process’s outcomes. The MRI images dataset was divided into four categories: ND, VMD, MD, and MOD. Over the MRI images dataset, the proposed fine-tuned ensemble, VGG16, MobileNet, and InceptionResNetV2, were evaluated by calculating the precision, sensitivity, F1-score, specificity, and accuracy.

Tables 7, 8, 9, and 10 demonstrated that the accuracy of the proposed fine-tuned ensemble had the highest precision, sensitivity, F1-score, specificity, and accuracy, at 95.69%, 95.62%, 95.64%, 98.54%, and 97.81% respectively. The InceptionResNetV2 had the lowest precision, sensitivity, F1-score, specificity, and accuracy, at 83.90%, 76.69%, 75.77%, 92.13%, and 88.11%, respectively.

For the ND class, the proposed ensemble achieved the highest precision, sensitivity, F1-score, specificity, and accuracy, at 98.57%, 97.34%, 97.95%, 99.53%, and 98.98%, respectively. The VGG16 had the lowest precision and specificity, at 90.36% and 96.67%, respectively. The InceptionRes-NetV2 had the lowest sensitivity, F1-score, and accuracy, at 73.40%, 81.49%, and 91.68%, respectively.

For the VMD class, the proposed ensemble achieved 99.84%, 100%, 99.92%, 99.95%, and 99.96% for precision, sensitivity, F1-score, specificity, and accuracy, respectively. The MobileNet achieved the highest precision, F1-score, specificity, and accuracy, at 100%. The proposed ensem-ble, VGG16, MobileNet, and InceptionResNetV2, achieved the highest sensitivity at 100%. The InceptionResNetV2 achieved the lowest precision, F1-score, and accuracy, at 99.37%, 99.69%, and 99.84%, respectively.

For the MD class, the proposed ensemble achieved 91.23%, 94.26%, 92.72%, 96.84%, and 96.17% for precision, sensitivity, F1-score, specificity, and accuracy, respectively. The Inception-ResNetV achieved the highest precision and specificity, at 92.45% and 98.89%, respectively. The proposed ensemble achieved the highest sensitivity, F1-score, and accuracy, at 94.26%, 92.72%, and 96.17%, respectively. The VGG16 achieved the lowest precision and specificity, at 81.76% and 93.47%, respectively. The InceptionResNetV achieved the lowest sensitivity, F1-score, and accu-racy, at 38.82%, 54.68%, and 83.36%, respectively.

For the MOD class, the proposed ensemble achieved 93.10%, 90.87%, 91.97%, 97.83%, and 96.13% for precision, sensitivity, F1-score, specificity, and accuracy, respectively. The proposed ensemble had the highest precision, F1-score, specificity, and accuracy, at 93.10%, 91.97%, 97.83%, and 96.13%, respectively. The InceptionResNetV achieved the lowest precision, F1-score, specific-ity, and accuracy, at 52.17%, 67.24%, 72.06%, and 77.54%, respectively. The VGG16 achieved the lowest sensitivity, at 76.92%.

**Table 7:** The results of the proposed ensemble on the test set of the MRI dataset for the four classes.

Class	Precision (%)	Sensitivity (%)	Specificity (%)	F1-score (%)	Accuracy (%)
**ND**	98.57	97.34	97.95	99.53	98.98
**VMD**	99.84	100.00	99.92	99.95	99.96
**MD**	91.23	94.26	92.72	96.84	96.17
**MOD**	93.10	90.87	91.97	97.83	96.13
**Average**	**95.69**	**95.62**	**95.64**	**98.54**	**97.81**

**Table 8:** The results of the VGG 16 on the test set of the MRI dataset for the four classes.

Class	Precision (%)	Sensitivity (%)	Specificity (%)	F1-score (%)	Accuracy (%)
**ND**	90.36	93.90	92.10	96.67	95.98
**VMD**	99.69	100.00	99.84	99.90	99.92
**MD**	81.76	83.99	82.86	93.47	91.02
**MOD**	82.90	76.92	79.80	94.89	90.51
**Average**	**88.68**	**88.70**	**88.65**	**96.23**	**94.36**

**Table 9:** The results of the MobileNet on the test set of the MRI dataset for the four classes.

Class	Precision (%)	Sensitivity (%)	F1-Score (%)	Specificity (%)	Accuracy (%)
**ND**	94.06	94.21	94.14	98.02	97.07
**VMD**	100.00	100.00	100.00	100.00	100.00
**MD**	88.21	77.95	82.76	96.36	91.60
**MOD**	77.57	87.02	82.02	91.89	90.70
**Average**	**89.96**	**89.79**	**89.73**	**96.57**	**94.84**

**Table 10:** The results of the InceptionResNetV2 on the test set of the MRI dataset for the four classes.

Class	Precision (%)	Sensitivity (%)	Specificity (%)	F1-score (%)	Accuracy (%)
**ND**	91.60	73.40	81.49	97.76	91.68
**VMD**	99.37	100.00	99.69	99.79	99.84
**MD**	92.45	38.82	54.68	98.89	83.36
**MOD**	52.17	94.55	67.24	72.06	77.54
**Average**	**83.90**	**76.69**	**75.77**	**92.13**	**88.11**

**Fig 3:**
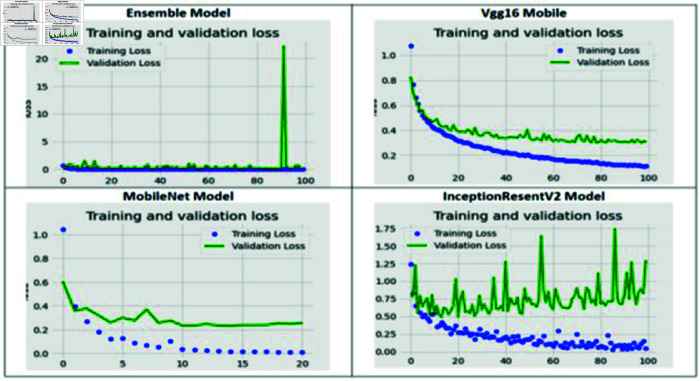
The four CNNs models’ loss vs. epoch numbers.

The training and validation loss of the proposed fine-tuned ensemble model, VGG16, MobileNet, and InceptionResNetV2, are shown in Fig 3. For the fine-tuned ensemble model, the training loss reached zero. Between the 20th and 90th epochs, the validation loss remained close to the training loss, indicating it was also near zero. This suggests that neither un-derfitting nor overfitting occurred in the proposed ensemble model. For the VGG16 model, both the training and validation loss decreased as the number of epochs increased. By the 100th epoch, the training loss approached zero, while the validation loss was below 0.4. In the case of the MobileNet model, the training loss became zero starting from the 10th epoch, and the validation loss remained low and stable. However, for the InceptionResNetV2 model, the validation loss was higher than the training loss, indicating that it experienced overfitting.

**Fig 4:**
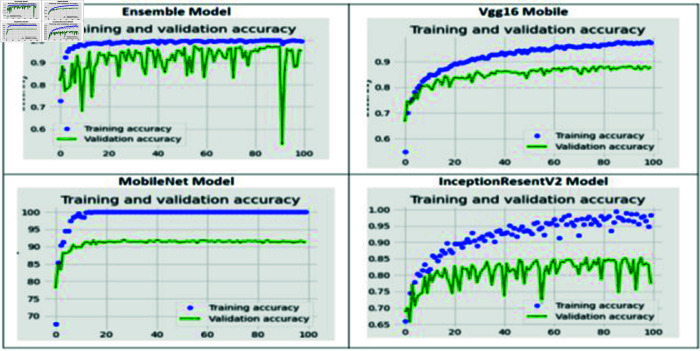
The ensemble and three CNN models’ accuracy vs. epoch numbers.

The training and validation accuracy of the proposed ensemble model, which includes VGG16, MobileNet, and InceptionResNetV2, is illustrated in Fig 4. For the fine-tuned ensemble model, after the 20th epoch, the training accuracy reached a steady state at 100%, indicating that the model was not overfitting. However, the validation accuracy showed some variability. For VGG16, both training and validation accuracy increased with the number of epochs. By the 100th epoch, the training accuracy was nearly 100%, while the validation accuracy approached 90%. In the case of MobileNet, after the 10th epoch, the training accuracy stabilized at 100%, and the validation ac-curacy remained consistently above 90%. On the other hand, InceptionResNetV2 displayed fluctuations in validation accuracy, suggesting that it was overfitting.

On the test set, four classes—ND, VMD, MD, and MOD—are represented by the confusion matrix for the proposed ensemble model and three different CNN models. 639 ND photos, 635 VMD images, 662 MD images, and 624 MOD images made up the test set.

**Fig 5:**
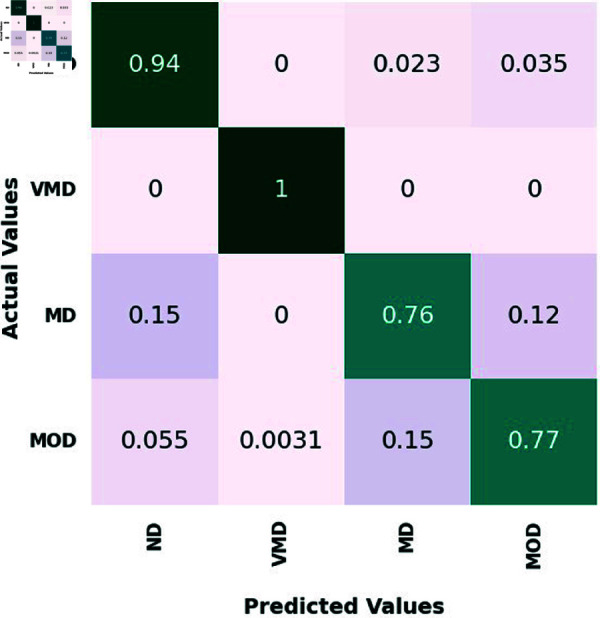
The confusion matrix of the proposed ensemble model.

**Fig 6:**
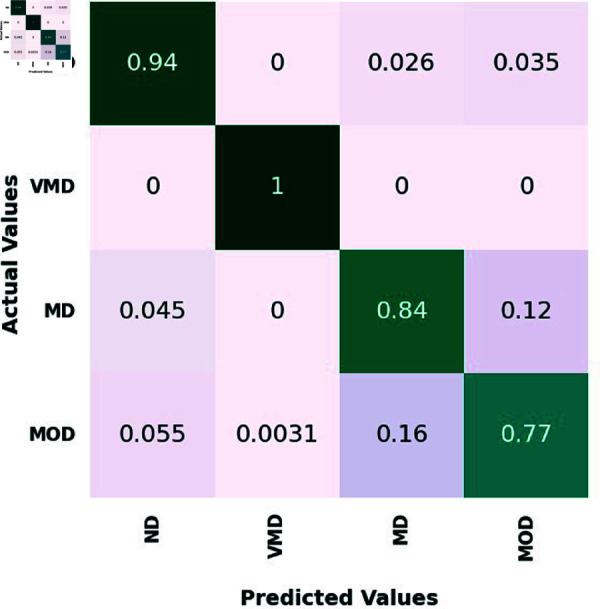
The confusion matrix of the InceptionResNetV2 model.

**Fig 7:**
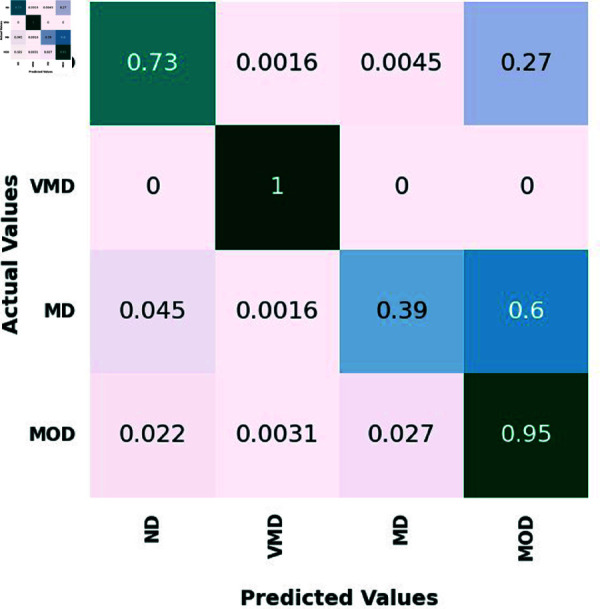
The confusion matrix of the MobileNet model.

**Fig 8:**
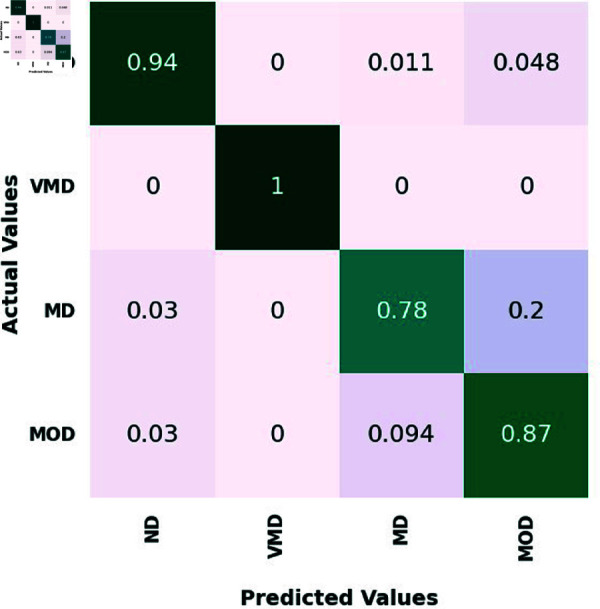
The confusion matrix of the VGG16 Model.

Figs 5, 6, 7, and 8, illustrate how the suggested ensemble mode exhibits outstanding diagnostic prowess across the spectrum of AD stages, from no disease ND to MOD. In the ND category, it accurately discerned 600 out of 639 cases, showcasing an exceptional ability to correctly identify individuals without the disease. In VMD stage, the Ensemble Model achieved perfection, with a 100% accuracy rate by precisely predicting all 635 cases. This highlights the model’s acute sensitivity to the subtlest signs of cognitive changes, affirming its potential for early detection and intervention. For the MD category, the Ensemble Model demonstrated near flawless performance, correctly classifying 556 out of 662, translating to a remarkable 83.9% accuracy rate.

In the MOD category, the Ensemble Model continued its streak of excellence, accurately pre-dicting 480 out of 624 cases, equivalent to an 76.9% accuracy rate. Comparatively, the VGG16, MobileNet, and InceptionResNetV2 models also demonstrated commendable performances, with particular strengths in various disease stages. The Ensemble Model, however, consistently outperformed the individual models, reinforcing its status as the winning approach. It combines the strengths of the individual models to deliver a superior pre-dictive tool, capable of operating with high accuracy across different severities of AD.

This Ensemble Model’s consistent high performance, especially in the challenging categories of VMD, MD, and MOD, showcases its comprehensive applicability and establishes it as a leading model in the medical image classification domain.

To further validate the strength of our proposed ensemble model, we conducted a second experiment using an external dataset - the Alzheimer’s Disease Neuroimaging Initiative (ADNI) dataset. This step ensured that the model can be effectively applied to real-world clinical data. The ADNI dataset comprises five classes: Control Normal (CN), Mild Cognitive Impairment (MCI), Early Mild Cognitive Impairment (EMCI), Late Mild Cognitive Impairment (LMCI), and Alzheimer’s disease (AD). The dataset consists of 1296 T1-weighted MRI scans, with the following distribution: CN (580 scans), MCI (233 scans), EMCI (240 scans), LMCI (72 scans), and AD (171 scans). Each scan produces a 3D picture of the brain with a resolution of 1.5 mm isotropic voxels.

**Fig 9:**
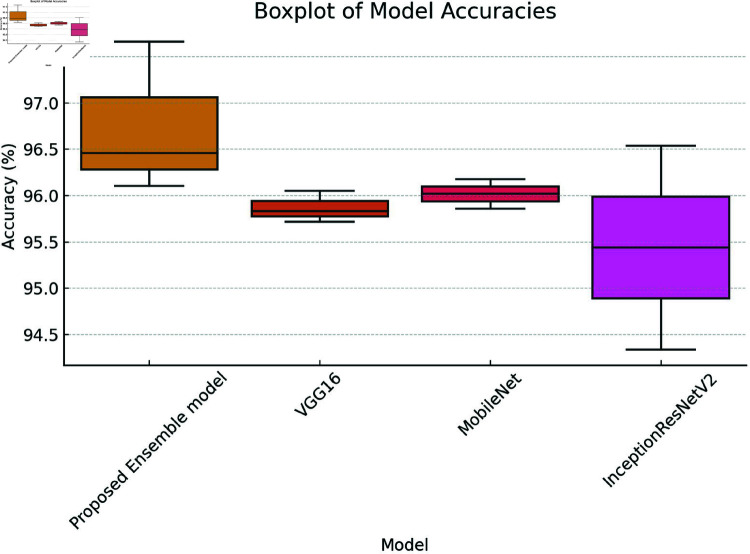
The four CNNs models’ loss vs. epoch numbers.

Tables 11, 12, 13, and 14 provided the average evaluation metrics for each model on the test set of the ADNI dataset.The proposed ensemble model achieved the highest average accuracy at 96.88%, outperforming VGG16 (95.88%), MobileNet (96.02%), and InceptionResNetV2 (95.44%). The proposed fine-tuned ensemble model had the best overall performance, with the highest accuracy 96.88%, specificity 97.82%, NPV 98.29%, and precision 90.14%. While the proposed ensemble had the highest accuracy, VGG16 recorded the highest recall and F1-score at 89.56% and 89.11% respectively. VGG16 also achieved the lowest false negative rate (FNR) at 10.44%. Therefore, the proposed fine-tuned ensemble model demonstrated the strongest overall performance across the evaluated metrics on the ADNI dataset test set. The performance of the proposed fine-tuned ensemble model,VGG16, MobileNet, and InceptionResNetV2, was evaluated using a boxplot shown in Fig 9. An ANOVA test was conducted on the ADNI dataset. The results for the classifiers based on the ADNI dataset features revealed the following values: the proposed ensemble model had a lower value of 96.10%, an upper value of 97.66%, and an average of 96.88%; VGG16 had a lower value of 95.72%, an upper value of 96.05 %, and an average of 95.88%; MobileNetV2 had a lower value of 95.86%, an upper value of 96.18%, and an average of 96.02%, and InceptionResNetV2 had a lower value of 94.3%, an upper value of 96.54%, and an average of 95.44%.

For the proposed ensemble model, the adjacent minimum and maximum values were 96.10% and 97.66%, respectively, with an average of 96.88 %. In the analysis of VGG16, the lower and upper adjacent values were 95.72% and 96.05%, respectively, resulting in a mean value of 95.88%. The MobileNetV2 classifier showed lower and upper adjacent points of 95.86% and 96.18%, respectively, with an average of 96.02%. For the InceptionResNetV2 test, the lower and upper adjacent values are 94.34% and 96.54%, leading to a mean value of 95.44%. The ANOVA results indicated that the proposed ensemble model demonstrated a strong performance on the ADNI dataset.

**Table 11:** The results of the proposed ensemble on the test set of the ADNI dataset.

	Accuracy	Specificity	FNR	NPV	Precision	Recall	F1-score
**1**	98.66	99.02	5.39	99.07	95.65	94.61	95.09
**2**	96.46	97.55	30.07	98.13	89.02	69.93	72.32
**3**	96.43	97.60	29.25	98.07	88.34	70.75	72.97
**4**	96.41	97.46	29.67	98.07	88.79	70.33	73.24
**5**	96.46	97.48	29.42	98.10	88.90	70.58	73.57
**Average**	**96.88**	**97.82**	**24.76**	**98.29**	**90.14**	**75.24**	**77.44**
**Confidence Interval**	[96.10 , 97.66 ]		

**Table 12:** The results of VGG16 on the test set of the ADNI dataset.

	Accuracy	Specificity	FNR	NPV	Precision	Recall	F1-score
**1**	95.64	96.62	12.45	96.76	89.23	87.55	88.15
**2**	95.75	96.72	10.90	96.84	89.50	89.10	89.18
**3**	96.16	97.18	8.93	97.06	89.13	91.07	90.02
**4**	96.05	97.11	10.13	97.02	87.75	89.87	88.65
**5**	95.83	96.90	9.80	96.79	89.10	90.20	89.57
**Average**	**95.88**	**96.90**	**10.44**	**96.90**	**88.94**	**89.56**	**89.11**
**Confidence Interval**	[95.72 , 96.05 ]		

**Table 13:** The results of MobileNet on the test set of the ADNI dataset.

	Accuracy	Specificity	FNR	NPV	Precision	Recall	F1-score
**1**	96.07	97.32	32.15	97.91	87.64	67.85	69.16
**2**	96.03	97.40	33.05	97.90	88.20	66.95	66.92
**3**	96.14	97.28	32.98	97.95	89.02	67.02	68.73
**4**	96.18	97.43	32.45	97.97	86.47	67.55	68.89
**5**	95.67	96.72	35.82	97.69	87.25	64.18	66.90
**Average**	**96.02**	**97.23**	**33.29**	**97.89**	**87.72**	**66.71**	**68.12**
**Confidence Interval**	[95.86 , 96.18 ]		

**Table 14:** The results of InceptionResNetV2 on the test set of the ADNI dataset.

	Accuracy	Specificity	FNR	NPV	Precision	Recall	F1-score
**1**	96.00	97.26	33.03	97.85	87.69	66.97	67.53
**2**	93.08	94.92	44.70	95.81	72.59	71.84	71.03
**3**	96.82	97.80	25.82	98.29	88.55	74.18	77.05
**4**	95.62	96.86	34.93	97.64	85.30	65.07	86.76
**5**	95.68	96.84	33.72	97.63	88.21	66.28	68.31
**Average**	**95.44**	**96.74**	**34.44**	**97.44**	**84.47**	**68.87**	**74.14**
**Confidence Interval**	[94.34 , 96.54 ]		

### Ablation study

In our methodology, we conducted an ablation study to understand the contribution of different components within the proposed ensemble model. The ablation study involved removing or "ablating" one of the base models and observing how the performance of the overall proposed model changed. This allowed us to determine the significance and impact that the removed model had on the overall performance of the proposed ensemble model. Tables 15, 16, and 17 showed case the results of ablation study. When the VGG16 model was removed from the ensemble, the accuracy of the proposed model was 97.19%. Similarly, when the InceptionResNetV2 model was removed, the accuracy of the proposed model was 97.49%. When the MobileNet model was removed, the accuracy of the proposed model was 97.21%.

Tables 4, 5, and 6 presented the performance of VGG16, MobileNet, and InceptionResNetV2 models on the testing set of the MRI images dataset after applying oversampling. The average accuracy for these models were 94.09%, 95.41%, and 91.15%, respectively.

The results demonstrated the effectiveness of the ensemble approach in improving the classification performance on the MRI image dataset with accuracy 97.93% as shown in Table 7, compared to the individual or two combinations of VGG16, MobileNet, and InceptionResNetV2 models.

**Table 15:** The results of the ensemble of MobileNet and InceptionResNetV2 on the test set of the MRI dataset.

	Accuracy	Specificity	FNR	NPV	Precision	Recall	F1-score
**1**	98.05	98.70	3.87	98.70	96.20	96.13	96.13
**2**	98.22	98.82	3.52	98.82	96.45	96.48	96.44
**3**	98.09	98.72	3.87	98.75	96.29	96.13	96.13
**4**	96.89	97.93	6.19	97.92	93.97	93.81	93.85
**5**	94.69	96.48	10.48	96.55	91.50	89.75	89.75
**Average**	**97.19**	**98.13**	**5.59**	**98.15**	**94.88**	**94.46**	**94.46**

**Table 16:** The results of the ensemble of MobileNet and VGG16 on the test set of the MRI dataset.

	Accuracy	Specificity	FNR	NPV	Precision	Recall	F1-score
**1**	94.94	96.65	10.02	96.79	91.93	89.98	90
**2**	98.44	98.96	3.10	98.96	96.88	96.90	96.88
**3**	97.70	98.46	4.62	98.48	95.50	95.38	95.37
**4**	98.54	99.02	2.90	99.02	97.08	97.10	97.08
**5**	97.85	98.56	4.33	98.58	95.83	95.67	95.70
**Average**	**97.49**	**98.33**	**4.99**	**98.37**	**95.44**	**95.01**	**95.01**

**Table 17:** The results of the ensemble of VGG16 and InceptionResNetV2 on the test set of the MRI dataset.

	Accuracy	Specificity	FNR	NPV	Precision	Recall	F1-score
**1**	98.50	99	3.01	99	97.01	96.99	97
**2**	98.50	99	3.01	99	97	96.99	96.99
**3**	92.27	94.86	15.26	95.26	87.65	84.74	84.28
**4**	98.42	98.95	3.15	98.94	96.84	96.85	96.84
**5**	98.38	98.92	3.21	98.92	96.77	96.79	96.77
**Average**	**97.21**	**98.14**	**5.53**	**98.22**	**95.05**	**94.47**	**94.37**

### The proposed ensemble’s result comparison with the recent research

This paper introduced a unique ensemble model that combined VGG16, MobileNet, and InceptionResNetV2, aimed at the multi-classification of AD. By fusing features from these models, we aimed to leverage their individual advantages:

1. VGG16: Excels at feature extraction due to its deep and uniform architecture, capturing fine details.
2. MobileNet: Lightweight and optimized for efficiency, contributing to computational feasibility and extracting diverse features.
3. InceptionResNetV2: Incorporates residual connections with Inception modules, allowing for efficient extraction of both local and global features.

By combining the features from these pre-trained models, the proposed approach leverages a diverse set of extracted characteristics, reducing the risk of overfitting and enhancing the ability to generalize across the MRI dataset. All the pre-trained individual feature vectors of VGG16, MobileNet, and InceptionResNetV2 are united into a single feature vector by utilizing a concatenate layer, thus refining the concatenated single feature vector. In Table 18, [12] utilized AlexNet. Although effective, AlexNet is relatively shallow when compared to architectures like VGG16, MobileNet, and InceptionResNetV2. This shallowness limits its ability to capture complex patterns in the data. [14] employed traditional ML methods, such as SVM, DT, RF, and KNN, which relied on handcrafted features. These approaches proved to be less effective than the deep feature extraction utilized in the proposed model. The Hybrid Classical–Quantum Approach mentioned in [18] heavily depended on specific configurations and may not generalize as effectively as the proposed ensemble model. Furthermore, in reference [17], although DEREx achieved 100% accuracy in binary classification, it struggled in multi-class scenarios, emphasizing the robustness of the proposed model in managing complex classification tasks. The proposed model likely benefited from advanced optimization techniques and a comprehensive training process, which enhanced its ability to learn from the data effectively. Overall, the combination of diverse deep learning architectures and the integration of their features were significant factors in the proposed model achieving an accuracy of 97.93%, surpassing existing methods. This model successfully balanced computational efficiency, feature diversity, and generalization capabilities. For accurate performance evaluation, McNemar’s test [24], which is a variation of the chi-squared test, was utilized as shown in Table 19. The initial step in McNemar’s test involves constructing a contingency table that outlines the successful and unsuccessful predictions made by the two chosen algorithms. According to this table, if (b >c ) (where ( b ) represents the number of instances in which algorithm A succeeds while algorithm B fails, and ( c ) represents the number of instances where algorithm B succeeds while algorithm A fails), then algorithm A is considered more effective than algorithm B.

For instance, in Table 19, both our proposed model and the model described in [12] correctly predict 587 images, while incorrectly predicting 10 images. Our model accurately predicts 40 images (denoted as b) that the model in [12] misclassifies. Conversely, our model incorrectly predicts 3 images (denoted as c) that the model in [12] classifies correctly. Since ( b >c ), it can be concluded that our proposed model is more effective than the algorithm presented in [12].

In the subsequent step, the z score is computed to quantify the difference in performance be-tween the two algorithms. A z score of 0 indicates acceptance of the null hypothesis (H0), which posits that there is no significant difference in performance between the algorithms. Conversely, if the z score deviates positively from 0, the alternative hypothesis (H1), which claims that there is a significant performance difference between the two algorithms, is accepted.

**Table 18:** Proposed model comparison with the recent methods.

Ref	Methodology	Performance	Dataset
**[12]**	Transfer learning and pre-trained AlexNet	91.70%	MRI Kaggle dataset
**[13]**	CNN	84.83% - 95.23%	MRI Kaggle dataset
**[14]**	SVM, DT, RF, and KNN	94%	OASIS dataset
	AlexNet+SVM and ResNet-50+SVM	94.8%	MRI Kaggle dataset
**[15]**	LMLS-SRC	85.54%	MRI Kaggle dataset
**[16]**	CNN+AlexNet	95%	MRI Kaggle dataset
**[17]**	DEREx	100% for binary classification	MRI Kaggle dataset
		91.49% for Multi-classification	
**[18]**	Hybrid classical–quantum transfer learning and GoogleNet/ResNet34	97%	MRI Kaggle dataset
**The Proposed Model**	VGG16, MobileNet, and InceptionResNetV2	97.93%	MRI Kaggle dataset

**Table 19:** McNemar’s test.

	[12] correct (91.70%)	[12] incorrect
**The Proposed Model correct (627) (97.93%)**	587	627-587=(40)
**The Proposed Model incorrect (13)**	3	10
	**[13] correct (95.23%)**	**[13] incorrect **
**The Proposed Model correct (627)**	609	627-609=(18)
**The Proposed Model incorrect (13)**	3	10
	**[14] correct (94.8%)**	**[14] incorrect **
**The Proposed Model correct (627)**	607	627-607=(20)
**The Proposed Model incorrect (13)**	3	10
	**[18] correct (97%)**	**[18] incorrect **
**The Proposed Model correct (627)**	620	627-620=(7)
**The Proposed Model incorrect (13)**	3	10
	**VGG16 correct (94.36%)**	**VGG16 correct **
**The Proposed Model correct (627)**	604	627-604=(23)
**The Proposed Model incorrect (13)**	3	10
	**MobileNet correct (94.84%)**	**MobileNet correct**
**The Proposed Model correct (627)**	607	627-607= (20)
**The Proposed Model incorrect (13)**	3	10
	**InceptionResNetV2 correct (88.11%)**	**InceptionResNetV2 incorrect **
**The Proposed Model correct (627)**	563	627-563= (64)
**The Proposed Model incorrect (13)**	3	10

**Table 20:** The P-Value test.

Metrics	Proposed Model	[12]	[13]	[14]	[15]	[17]	[18]
Accuracy (%)	97.93	91.7	95.23	94.1	87.9	96.89	95
Precision (%)	95.94	91.50	96	–	88.71	–	91
Sensitivity (%)	95.89	93.70	95	91.75	89.25	–	94
Specificity (%)	98.04	96	–	97.5	86.53	–	–
F1-Score (%)	96.36	–	95.27	–	88.44	96.83	87
P-Value	1	0.0483	0.3671	0.2870	0.00	0.4999	0.0619

The Probability Value (P-Value) is a statistical measure utilized in hypothesis testing to assess the likelihood that the observed data occurred by random chance, assuming that the null hypothesis is true. If the P-Value *leq* 0.05, it suggests that there is a statistically significant difference. This implies that the proposed model is likely to perform either better or worse than the reference model with confidence. Conversely, if the P-Value , it indicates that there is no statistically significant difference. In this case, the performance differences may be attributed to random variation.

The P-Value as shown in Table 20 between the proposed model and [12] is 0.0483, indicating that the difference is statistically significant for at least one metric, such as accuracy or precision. This suggests that the proposed model likely provides a meaningful improvement over reference [12]. In comparison to [13], the P-Value is 0.3617, which indicates that the difference is not statistically significant. Therefore, there is no strong evidence to conclude whether the proposed model is better or worse than [13]. The P-Value between the proposed model and [14] is 0.2870, also indicating that the difference is not statistically significant. Thus, the performance of the proposed model and [14] is similar, falling within the margin of random variation. For [15], the P-Value is 0.00, which signifies a statistically significant difference. Consequently, the proposed model significantly outperforms or underperforms [15], and based on accuracy and other metrics, it is likely to outperform it. The P-Value between the proposed model and [17] is 0.4999, suggesting that the difference is not statistically significant. Hence, both models exhibit similar performance without a notable advantage for the proposed model. Finally, the P-Value between the proposed model and [18] indicates a borderline difference, slightly above the threshold of statistical significance. While it is not conclusively significant, the proposed model may perform slightly better than [18], warranting further investigation.

Overall, the proposed model shows statistically significant improvements over [12] and [15]. Performance differences with [13,14,17,18] are not significant, suggesting comparable results.

## Conclusion

This paper introduced a fine-tuned ensemble model based on VGG16, MobileNet, and InceptionResNetV2 for multi-classification of AD using MRI images. Each one of VGG16, MobileNet, and InceptionResNetV2 suffers from limitations when it comes to variations in the shape and texture of the input image. The ensemble integrates the strengths of these three CNN models to address their individual limitations in handling variations in shape and texture of input images, leading to high classification accuracy. The proposed ensemble will assist radiologists by simplifying the diagnostic process, promoting early detection, and allowing for prompt treatment of AD. We extracted features from AD MRI scans using three DL models: VGG16, MobileNet, and InceptionResNetV2. The ImageNet dataset was initially used to pre-train the models. The layers were then flattened into a vector by all of the pre-trained models using GlobalAveragePooling2D to determine the average value for each of the input channels. The different vectors were then combined into a single vector with characteristics using the concatenate layer. Finally, the concatenated single feature vector on the MRI images dataset was fine-tuned by using six layers. The MRI image dataset was pre-processed using techniques for data augmentation, normalization, scaling. The model achieved average scores of 95.94% precision, 95.89% sensitivity, 98.04% specificity, 96.36% F1-score, and 97.93% accuracy, outperforming recent DL models. The paper highlights the ensemble’s potential to aid in early AD detection, reducing effort, time, and cost for pathologists. However, the challenge of processing speed remains. Future work will explore the model’s application to other diseases, utilize hyper-optimization algorithms for refining hyper-parameters, and implement a Reinforcement Learning-based Transformer Network (RTN) to improve MRI image quality, which is crucial for accurate AD detection.

## References

[1] Liu S, Liu S, Cai W, Che H, Pujol S, Kikinis R, et al. Multimodal neuroimaging feature learning for multiclass diagnosis of Alzheimer’s disease. IEEE Trans Biomed Eng 2015;62(4):1132–40. doi: 10.1109/TBME.2014.2372011 25423647 PMC4394860

[2] Beheshti I, Demirel H, Alzheimer’s Disease Neuroimaging Initiative. Feature-ranking-based Alzheimer’s disease classification from structural MRI. Magn Reson Imaging 2016;34(3):252–63. doi: 10.1016/j.mri.2015.11.009 26657976

[3] Afzal S, Maqsood M, Nazir F, Khan U, Aadil F, Awan KM, et al. A data augmentation-based framework to handle class imbalance problem for Alzheimer’s stage detection. IEEE Access. 2019;7:115528–39. doi: 10.1109/access.2019.2932786

[4] Wang Y-Q, Jia R-X, Liang J-H, Li J, Qian S, Li J-Y, et al. Dementia in China (2015-2050) estimated using the 1 10.1111/ggi.13778 31535462

[5] Giorgio J, Landau SM, Jagust WJ, Tino P, Kourtzi Z, Alzheimer’s Disease Neuroimaging Initiative. Modelling prognostic trajectories of cognitive decline due to Alzheimer’s disease. Neuroimage Clin. 2020;26:102199. doi: 10.1016/j.nicl.2020.102199 32106025 PMC7044529

[6] Lessov-Schlaggar CN, Del Rosario OL, Morris JC, Ances BM, Schlaggar BL, Constantino JN. Adaptation of the clinical dementia rating scale for adults with down syndrome. J Neurodev Disord 2019;11(1):39. doi: 10.1186/s11689-019-9300-2 31842726 PMC6912998

[7] Umbach G, Kantak P, Jacobs J, Kahana M, Pfeiffer BE, Sperling M, et al. Time cells in the human hippocampus and entorhinal cortex support episodic memory. Proc Natl Acad Sci U S A 2020;117(45):28463–74. doi: 10.1073/pnas.2013250117 33109718 PMC7668099

[8] 2019 Alzheimer’s disease facts and figures. Alzheimer’s & Dementia. 2019;15(3):321–87

[9] Lee S, Lee H, Kim KW, Alzheimer’s Disease Neuroimaging Initiative. Magnetic resonance imaging texture predicts progression to dementia due to Alzheimer disease earlier than hippocampal volume. J Psychiatry Neurosci 2020;45(1):7–14. doi: 10.1503/jpn.180171 31228173 PMC6919919

[10] Moradi E, Pepe A, Gaser C, Huttunen H, Tohka J, Alzheimer’s Disease Neuroimaging Initiative. Machine learning framework for early MRI-based Alzheimer’s conversion prediction in MCI subjects. Neuroimage. 2015;104:398–412. doi: 10.1016/j.neuroimage.2014.10.002 25312773 PMC5957071

[11] Bron EE, Smits M, van der Flier WM, Vrenken H, Barkhof F, Scheltens P, et al. Standardized evaluation of algorithms for computer-aided diagnosis of dementia based on structural MRI: the CADDementia challenge. Neuroimage. 2015;111:562–79. doi: 10.1016/j.neuroimage.2015.01.048 25652394 PMC4943029

[12] M. Ghazal T, Abbas S, Munir S, A. Khan M, Ahmad M, F. Issa G, et al. Alzheimer disease detection empowered with transfer learning. Comput Mater Continua 2022;70(3):5005–19. doi: 10.32604/cmc.2022.020866

[13] Murugan S, Venkatesan C, Sumithra MG, Gao X-Z, Elakkiya B, Akila M, et al. DEMNET: a deep learning model for early diagnosis of Alzheimer diseases and dementia from MR images. IEEE Access. 2021;9:90319–29. doi: 10.1109/access.2021.3090474

[14] Mohammed BA, Senan EM, Rassem TH, Makbol NM, Alanazi AA, Al-Mekhlafi ZG, et al. Multi-method analysis of medical records and MRI images for early diagnosis of dementia and Alzheimer’s disease based on deep learning and hybrid methods. Electronics, vol. 10, 2021.

[15] Liu R, Li G, Gao M, Cai W, Ning X. Large margin and local structure preservation sparse representation classifier for Alzheimer’s magnetic resonance imaging classification. Front Aging Neurosci. 2022;14:916020. doi: 10.3389/fnagi.2022.916020 35693338 PMC9177229

[16] Fu’adah YN, Wijayanto I, Pratiwi NKC, Taliningsih FF, Rizal S, Pramudito MA. Automated classification of Alzheimer’s disease based on MRI image processing using Convolutional Neural Network (CNN) with AlexNet architecture. J Phys: Conf Ser 2021;1844(1):012020. doi: 10.1088/1742-6596/1844/1/012020

[17] De Falco I, De Pietro G, Sannino G. A two-step approach for classification in Alzheimer’s disease. Sensors (Basel) 2022;22(11):3966. doi: 10.3390/s22113966 35684587 PMC9183018

[18] Shahwar T, Zafar J, Almogren A, Zafar H, Rehman AU, Shafiq M, et al. Automated detection of Alzheimer’s via hybrid classical quantum neural networks. Electronics, vol. 11, 2022.

[19] Dubey, S. [cited 2021 Apr]. Available from: https://www.kaggle.com/tourist55/alzheimers-dataset-4-class-of-images

[20] Simonyan K, Zisserman A. Very deep convolutional networks for large-scale image recognition. arXiv preprint 2014

[21] Howard A, Zhu M, Chen B, Kalenichenko D, Wang W. MobileNets: efficient convolutional neural networks for mobile vision applications. arXiv preprint 2017

[22] Bilal M, Maqsood M, Yasmin S, Hasan NU, Rho S. A transfer learning-based efficient spatiotemporal human action recognition framework for long and overlapping action classes. J Supercomput 2021;78(2):2873–908. doi: 10.1007/s11227-021-03957-4

[23] Mohammed A, Kora R. An effective ensemble deep learning framework for text classification. J King Saud Univ - Comput Inf Sci 2022;34(10):8825–37. doi: 10.1016/j.jksuci.2021.11.001

[24] Bostanci B, Bostanci E. An evaluation of classification algorithms using Mc Nemar’s test. In Proceedings of Seventh International Conference on Bio-inspired Computing: Theories and Applications (BIC-TA 2012). Adv Intell Syst Comput. 2013;201:15–26.

